# Interethnic diversity of the CD209 (rs4804803) gene promoter polymorphism in African but not American sickle cell disease

**DOI:** 10.7717/peerj.799

**Published:** 2015-02-24

**Authors:** Jenelle A. Noble, Kimberley C. Duru, Aldiouma Guindo, Li Yi, Ikhide G. Imumorin, Dapa A. Diallo, Bolaji N. Thomas

**Affiliations:** 1 Department of Biomedical Sciences, College of Health Sciences and Technology, Rochester Institute of Technology, Rochester, NY, USA; 2 Centre de Recherche et de Lutte contre la Drepanocytose, Bamako, Mali; 3 School of Statistics, Shanxi University of Finance and Economics, Shanxi, China; 4 Animal Genetics and Genomics Lab, Office of International Programs, Cornell University, Ithaca, NY, USA

**Keywords:** Sickle cell disease, Africa, African Americans, Caucasians, CD209, Diversity, Interethnic, Populations, Co-morbidities

## Abstract

Elucidating the genomic diversity of CD209 gene promoter polymorphism could assist in clarifying disease pathophysiology as well as contribution to co-morbidities. CD209 gene promoter polymorphism has been shown to be associated with susceptibility to infection. We hypothesize that CD209 mutant variants occur at a higher frequency among Africans and in sickle cell disease. We analyzed the frequency of the CD209 gene (rs4804803) in healthy control and sickle cell disease (SCD) populations and determined association with disease. Genomic DNA was extracted from blood samples collected from 145 SCD and 231 control Africans (from Mali), 331 SCD and 379 control African Americans and 159 Caucasians. Comparative analysis among and between groups was carried out by polymerase chain reaction-restriction fragment length polymorphism (PCR-RFLP). Per ethnic diversification, we found significant disparity in genotypic (23.4% versus 16.9% versus 3.2%) and allelic frequencies (48.7% versus 42.1% versus 19.8%) of the homozygote mutant variant of the CD209 (snp 309A/G) gene promoter between Africans, African Americans and Caucasians respectively. Comparative evaluation between disease and control groups reveal a significant difference in genotypic (10.4% versus 23.4%; *p* = 0.002) and allelic frequencies (39.7% versus 48.7%; *p* = 0.02) of the homozygote mutant variant in African SCD and healthy controls respectively, an observation that is completely absent among Americans. Comparing disease groups, we found no difference in the genotypic (*p* = 0.19) or allelic (*p* = 0.72) frequencies of CD209 homozygote mutant variant between Africans and Americans with sickle cell disease. The higher frequency of CD209 homozygote mutant variants in the African control group reveals a potential impairment of the capacity to mount an immune response to infectious diseases, and possibly delineate susceptibility to or severity of infectious co-morbidities within and between groups.

## Introduction

Sickle cell disease (SCD) is an inherited multisystem disorder, characterized by chronic hemolytic anemia, vaso-occlusive crises and several other disease outcomes such as acute chest syndrome, bacteremia, leg ulcers and priapism (Bunn, 1997; Benkerrou et al., 2002). SCD has shown marked variability in severity between individuals, with evidence of extensive differences in both clinical and disease haplotypes, with a global distribution, especially in sub-Saharan Africa, Middle East, parts of the Indian subcontinent, and Americans with an African or Caribbean descent (Hassell, 2010; Piel et al., 2013; Bandeira et al., 2014; Saraf et al., 2014; Thakur et al., 2014). SCD occurs in patients that are homozygous for the hemoglobin S gene, produced by a defective *β*-globin gene on chromosome 11 and has also been defined as resulting from compound heterozygosity for hemoglobin S and another *β*-globin chain abnormality (typically hemoglobin C or *β*-thalassemia), with *α*-thalassemia serving as a modifier of the clinical manifestations (Weatherall, 2010; Saraf et al., 2014). Patients commonly require red cell transfusions to manage complications, with alloimmunization a common occurrence (Charache, Bleecker & Bross, 1983; Rosse, Gallagher & Kinney, 1990; Tatari-Calderone et al., 2013) leaving such multiply transfused patients at risk for delayed hemolytic transfusion reactions (Piomelli, 1985; Petz et al., 1997; Taylor et al., 2008; Yazdanbakhsh, Ware & Noizat-Pirenne, 2012), development of autoimmune hemolytic anemia.

Infectious pathogens are a threat to those individuals with SCD, particularly children, that are prone to frequent and severe attacks (Overturf, 1999; Halasa et al., 2007; Szczepanek et al., 2013). For children in endemic countries, with very high circulating immune complexes due to constant exposure to multiple pathogenic stimuli, the added burden of these co-morbidities can severely impact immune response and survival (Thomas et al., 2012a). Recent reports showing high mortality rates post-vaccination in transgenic animals demonstrates that a dysregulated immune response might be responsible for such mortality and could be a major drawback to the current push to vaccinate (Adamkiewicz et al., 2003; McCavit et al., 2011; Szczepanek et al., 2013). In fact, other reports have shown that there is an over-stimulation of pro-inflammatory cytokines in sickle cell disease patients, which might be be related to vaso-occlusion (Makis, Hatzimichael & Bourantas, 2000; Pathare et al., 2004; Steinberg, 2006; Conran, Franco-Penteado & Costa, 2009; Qari, Dier & Mousa, 2012; Bandeira et al., 2014). In fact, this hyperstimulation has been associated with sickle cell haplotype in Brazil, and as such is the obvious consequence of worsening immune response to secondary infectious pathogens or co-morbidities of infection.

Recently published data have shown that there are wide differences in *Plasmodium falciparum* infection rates and multiplicity of infection between children who are carriers of the sickle cell trait (hemoglobin AS) and those patients that possess the normal hemoglobin (HbAA) gene (Williams et al., 2005; Kreuels et al., 2010; Gong et al., 2012; Taylor, Parobek & Fairhurst, 2012; Gong et al., 2013). In addition, extensive differences in genomic diversity of endothelial nitric oxide synthase (eNOS) genes, that had been reported to bear clinical significance on sickle cell pathogenesis, has been reported between Africans and African Americans (Thomas et al., 2013). These polymorphisms have been shown to be potential modifiers of clinical disease, with significant differences reported between Indian and African sickle cell disease patients (Nishank et al., 2013; Thakur et al., 2014), and these differences could be potentially linked to disease haplotype. These interethnic differences can be attributed to the introduction of single nucleotide polymorphisms over a very long period, which can ultimately influence gene expression, protein structure and potentially function. Therefore, single nucleotide polymorphisms located in certain promoter regions can affect transcription thereby altering variability in the immune response, and contributing to disease susceptibility or host resistance (Sakuntabhai et al., 2005). Despite the fact that African Americans can trace their ancestry to sub-Saharan Africa, recombination and genetic diversity in the African American gene pool has facilitated the introduction of single nucleotide polymorphisms leading to differing immune response to infectious pathogens, such as malaria and tuberculosis (Thomas et al., 2005; Jallow et al., 2009; Thomas et al., 2012b; Noumsi et al., 2011; Hansson et al., 2013), and demonstrated in an Afro-Brazilian population (Covas et al., 2007; Dettogni et al., 2013) sharing phenotypic and genotypic similarity with African Americans. In addition, they are exposed to different groups of infectious agents compared to their African counterparts, which in turn directs immune system development, as shown in complement receptor-1 (CR1) polymorphisms in malaria-endemic and non-endemic populations (Thomas et al., 2005). These phenomena would undergo a similar diversification in the sickle cell disease population as well.

One of the most common immunogenetic markers, usually evaluated for immune system response and susceptibility to infectious pathogens is dendritic cell-specific ICAM-3 grabbing non-integrin (DC-SIGN) encoded by CD209. It assists in the migration dendritic cells on endothelium as well as enabling the activation of signal transduction pathways (Rappocciolo, Jenkins & Hensler, 2006; Dettogni et al., 2013). They are targets for pathogens, seeking to impair the immune response in early infection, and are known to recognize diverse pathogens, with reports showing association between CD209 gene polymorphisms and infectious agents (Mummidi et al., 2001; Martin et al., 2004). The guanine (G) to adenine (A) transition within the gene promoter (SNP-336 A/G; rs4804803) polymorphism has shown the most significance, demonstrating association with susceptibility to HIV, tuberculosis, leishmaniasis and dengue (Tailleux, Schwartz & Herrmann, 2003; Tassaneetrithep, Burgess & Granelli-Piperno, 2003; Van Kooyk, Appelmelk & Geijtenbeek, 2003; Martin et al., 2004; Sakuntabhai et al., 2005; Barreiro, Neyrolles & Babb, 2006). Due to the interaction between malaria and sickle cell disease, the possibility of imposing selection pressures, leading to changes in allele frequencies that can exacerbate or ameliorate outcome of disease co-morbidities exists (Thomas et al., 2012a; Thomas et al., 2012b). We have shown that there is an extensive diversity in the ethnogenomic distribution of endothelial nitric oxide synthase (eNOS) polymorphisms (Thomas et al., 2013). Despite reports to the contrary, we have also demonstrated that endothelin-1 polymorphisms, rather than eNOS, are the most important in African SCD (Thakur et al., 2014). Therefore, since infections are common occurrences in SCD, there is a need to characterize the genomic diversity as well as haplotype frequency of immunogenetic markers, thereby clarifying their contributions to infectious disease susceptibility or co-morbidities. To this end, we examined the genotypic and allelic frequency of CD209 gene promoter polymorphism (SNP-336 A/G; rs4804803) in control groups (Africans versus African Americans versus Caucasians) and between sickle cell disease populations (African versus American). We conducted our analyses using a polymerase chain reaction-restriction fragment length polymorphism (PCR-RFLP) assay.

## Materials and Methods

### Subjects

This study encompasses sickle cell disease patients (cases) and control groups (Africans versus African Americans), as well as diverse ethnic groups (Africans, African Americans and Caucasians). The African portion was conducted at the Centre de Recherche et de Lutte contre la Drepanocytose (CRLD), a sickle cell disease treatment and referral center in Bamako, Mali. This study was approved by the Institutional Review Board (IRB), Rochester Institute of Technology in addition to the original approval granted by the National Ethical Review Board in Mali. Inclusion criteria include diagnosis with sickle cell disease and presentation during crisis or during regular follow-up. Sickle cell disease and control group demographic data has been described previously (Thakur et al., 2014). Briefly, African sickle cell disease group consists of 51.5% males and 48.4% females (mean age: 21 years; range: 1-51 years), and predominantly of the Bambaran tribe. Healthy population controls comprised of family members or those recruited by word of mouth, able to provide informed consent and without a diagnosis of sickle cell disease. In the United States, control groups are African American and Caucasian self-identified individuals, recruited from Shreveport, Louisiana. African American sickle cell disease patients were recruited as part of the National Institute of Health-funded Cooperative Study of Sickle Cell Disease (CSSCD).

### Samples and genomic DNA extraction

Discarded EDTA-anticoagulated blood samples, from 376 subjects (145 sickle cell disease patients and 231 controls) were spotted onto filter papers (GE Healthcare Sciences, Piscataway, New Jersey, USA) and genomic DNA samples extracted from the dried, spotted samples with the Qiagen Blood Mini Kit (Qiagen Inc., Valencia, California, USA), with some changes to the manufacturer’s instruction (Thakur et al., 2014). Final elution volume was 100 µl and DNA samples were stored at −20 °C until further analysis. Genomic DNA samples from African American sickle cell disease patients as well as African American and Caucasian controls were gratefully provided (Betty Pace, Georgia Regents University and Joann Moulds, Grifols USA respectively).

### Genotyping for CD209 single nucleotide polymorphism

To genotype for the single nucleotide polymorphisms of the CD209 gene promoter, we utilized a previously published mis-matched primer, designed to artificially introduce a restriction site (Sakuntabhai et al., 2005) and PCR assay (Dettogni et al., 2013), with a slight modification to the protocol. The primer sequences are 5′-GGATGGTCTGGGGTTGACAG-3 (forward reaction) and 5′-ACTGGGGGTGCTACCTGGC-3′ (reverse reaction). One µl of genomic DNA served as the template for PCR amplification, with conditions optimized to 25 µl final volume and amplified using the Lucigen EconoTaq Plus Green 2X Master Mix PCR system (Lucigen Corporation, Middleton WI), as described previously (Thomas et al., 2012a), and PCR cycling parameters as published (Sakuntabhai et al., 2005). Amplified PCR products (5 µl) was examined on a 2% (w/v) agarose gel and photographed. Positive amplification yielded products of 150 bp, with size estimated with a TriDye 100 bp DNA ladder (New England Biolabs, Boston, Massachusetts, USA).

### Restriction fragment length polymorphism assay

We utilized the *MscI* (New England Biolabs, Boston, Massachusetts, USA) restriction endonuclease for restriction fragment length polymorphism analysis of CD209 (DC-SIGN 336A/G) gene promoter variants. 10 µl of PCR product was mixed with 0.5 µl of enzyme (5,000 U/ml), 5 µl of 1X CutSmart buffer and incubated at 37 °C for 1 h. Digested products were analyzed on an ethidium bromide-stained agarose gel, and band analysis carried out with a Doc-It LS Image Analysis Software (UVP Life Sciences, Upland, California, USA). Restriction analysis was conducted by two investigators anonymously and 50% of amplified products subjected to repeat digestion (3rd investigator), with 100% concordance. Homozygote wild type variants (−336A/A) were undigested (150 bp) while homozygote mutant variants (−336G/G) produced bands of 131 and 19 bp (Supplemental Information 1).

### Statistical analysis

Genotypic and allelic frequencies were determined with a simple PERL script, as described previously (Thakur et al., 2014). Differences in genotype and allele frequencies between populations were assessed by chi-square tests, while differences between sickle cell disease and controls were assessed by odds ratio. Tests for deviation from Hardy-Weinberg equilibrium (HWE) were performed, with SNP’s rejected based on the recommended threshold of *p* < 0.05 in control individuals. Briefly, we calculated the number of alleles and observed genotypes, and compared observed numbers of genotypes with that expected under HWE, where the latter are computed on the basis of allele frequencies estimated from the genotype frequencies (null hypothesis, H0). For observed numbers, the relative cell frequencies are the estimates of the genotype probabilities (alternative hypothesis, H1). For the comparison between observed and expected numbers of genotypes, likelihood ratio chi-square is computed. Power calculations were computed using the Vanderbilt University PS program http://biostat.mc.vanderbilt.edu/wiki/Main/PowerSampleSize.

## Results

**Figure 1:**
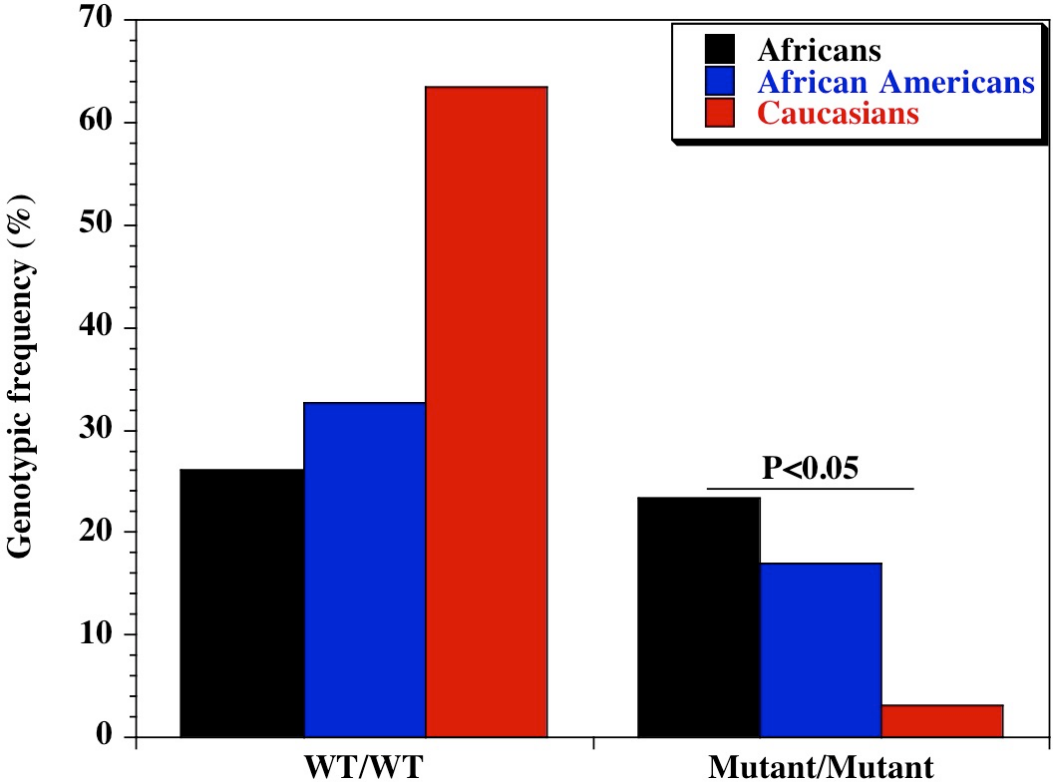
Genotypic distribution of CD209 gene promoter polymorphism (SNP-336 A/G; rs4804803) in Caucasian, African American and African healthy controls. Amplified PCR products were digested with *MscI* restriction endonuclease (Fisher Scientific, Waltham, Massachusetts, USA), and expressed on a 2% ethidium bromide-stained agarose gel. Homozygote wild type variant (snp-336A) showed no digestion (150 bp); homozygote mutant variant (snp-336G) produced two bands (131 and 19 bp) on digestion (lower band size not shown). Marker: 100 bp ladder, where the 500 bp band stains most intensely (New England Biolabs, Ipswich, Massachusetts, USA). Black bars: Africans; blue bars: African Americans; red bars: Caucasians.

We found a significant difference in the genetic diversity of the promoter variant of CD209 (DC-SIGN1-336A/G; rs4804803) gene polymorphism in different populations. Genotypic frequencies of 23.4%, 16.9% and 3.2% were observed for the homozygote mutant variant between Africans, African Americans and Caucasians respectively (Table 1). Similar findings were made for the allelic frequencies of the homozygote mutant variants (48.7%, 42.1% and 19.8% respectively), with a significant difference in the genotypic and allelic frequencies (*P* < 0.05) of CD209 gene promoter variant between all population groups. Surprisingly, the homozygote mutant variant (GG) is almost absent among Caucasians (3.2%; Fig. 1). The genotypic and allelic frequencies of the homozygote mutant variant (snp-336GG) had the highest frequency among Africans (23.4% and 48.7% respectively). The wild type and heterozygote variants (AA and AG), that are necessary to facilitate dendritic cell activation and function during immune response, occurred at higher frequencies among Caucasians (96.8%) and African Americans (83.1%), compared to the 76% among healthy African controls.

**Table 1:** Genotypic and allelic frequency of CD209 gene promoter polymorphism in diverse populations. Genotypic and allelic frequencies of the CD209 gene promoter (rs4803803) polymorphism, determined among African (*n* = 244), African American (*n* = 379) and Caucasian (*n* = 159) healthy controls. Healthy control populations are individuals without sickle cell disease (HbAA). Africans were recruited from Bamako, Mali; African American and Caucasian populations were recruited from Shreveport, Louisiana. Odds ratio was calculated by Fisher’s two-tailed exact test. *P* value <0.05 was considered significant.

Polymorphism	Genotype	Ethnic groups			Chi square	*P*-value
		African *n* = 231 (%)	African American *n* = 379 (%)	Caucasian *n* = 159 (%)		
CD209 (rs4084803)	A/A	60 (26.0)	124 (32.7)	101 (63.5)	62.97	2.12E−14
	A/G	117 (50.6)	191 (50.4)	53 (33.3)	14.91	5.78E−04
	G/G	54 (23.4)	64 (16.9)	5 (3.2)	29.13	4.72E−07
		**Allelic diversity**				
	**Allele**	***n*** **=** **462** (%)	***n*** **=** **758** (%)	***n*** **=** **318** (%)	**Chi square**	***P***-**value**
CD209 (rs4804803)	A	237 (51.3)	439 (57.9)	255 (80.2)	70.09	6.03E−16
	G	225 (48.7)	319 (42.1)	63 (19.8)	70.09	6.03E−16

**Figure 2:**
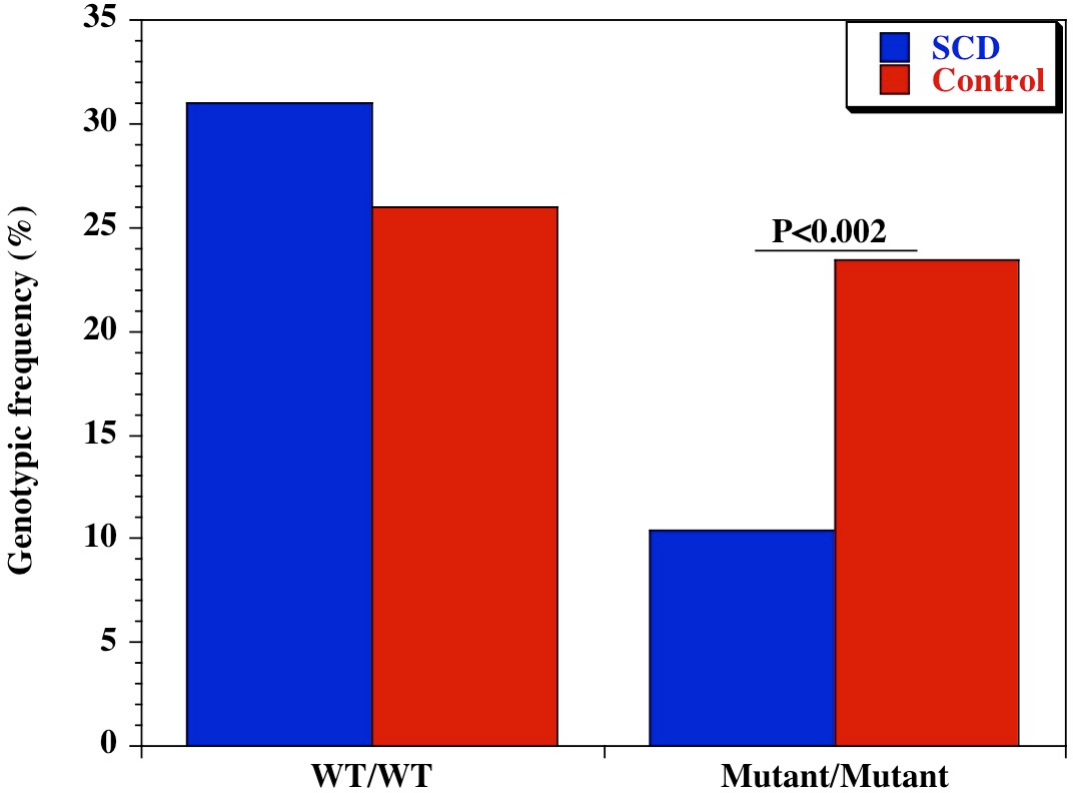
Genotypic frequency of CD209 gene promoter polymorphism (SNP-336 A/G; rs4804803) among African sickle cell disease and control groups. Amplified PCR products were digested with *MscI* restriction endonuclease (Fisher Scientific, Waltham, Massachusetts, USA), and expressed on a 2% ethidium bromide-stained agarose gel. Homozygote wild type variant (snp-336A) showed no digestion (150 bp); homozygote mutant variant (snp-336G) produced two bands (131 and 19 bp) on digestion (lower band size not shown). Marker: 100 bp ladder, where the 500 bp band stains most intensely (New England Biolabs). Blue bars-sickle cell disease; red bars-control groups.

**Figure 3:**
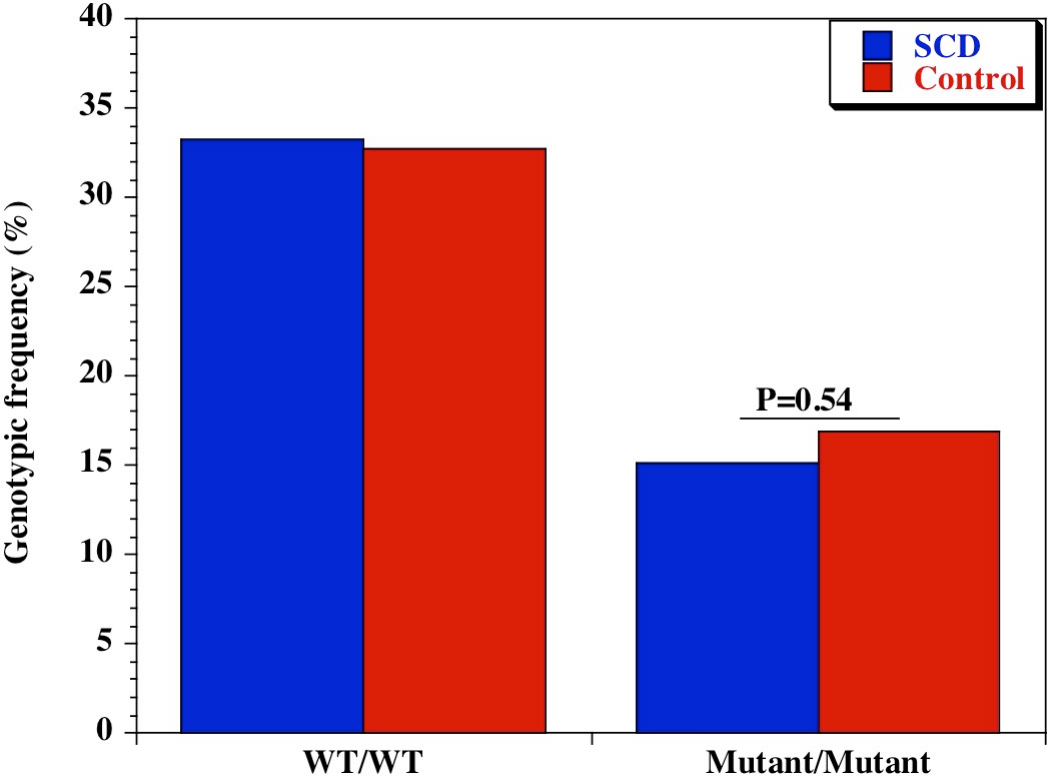
Genotypic frequency of CD209 gene promoter polymorphisms (SNP-336 A/G; rs4804803) *among* African American sickle cell disease and control groups.

We also examined the diversity of CD209 (snp 336A/G) gene promoter polymorphisms between sickle cell disease and healthy control groups in Africa and United States. There was an extensive and significant difference in the genotypic (Fig. 2 and Table 2) frequency of the CD209 mutant variant (snp 336G/G) between sickle cell disease and control populations in Africa (*P* = 0.002). Surprisingly, this was not the case between sickle cell disease and control populations recruited from the United States (Fig. 3) (*P* = 0.54). In addition, the mutant variant has a higher frequency among healthy control groups than sickle cell patients (23.4% versus 10.4% respectively) in Africa, but no difference in the United States (16.9% versus 15.1% for controls and cases respectively). Similar observation was made for the allelic frequencies between controls and cases in Africa and United States (Table 3). The SNP effect is insignificant between American sickle cell disease and controls, but significant among Africans, with a 40% power from our analysis.

**Table 2:** Genotypic frequency of CD209 polymorphisms between sickle cell and control groups. Genotypic frequencies of the CD209 gene promoter (rs4803803) polymorphism, determined among African and American sickle cell disease and control groups. Sickle cell disease (HbSS) populations were recruited from Bamako, Mali (African) and Augusta GA (American). Control populations are individuals without sickle cell disease, and were recruited from Mali (African) and Shreveport LA (American). A/G denotes the alleles at the CD209 locus. Odds ratio was calculated by Fisher’s two-tailed exact test. *P* value <0.05 was considered significant.

		African			
Polymorphism	Genotype	SCD: *n* = 145 (%)	Controls: *n* = 231 (%)	Odds ratio (95% CI)	*P*-value
CD209 (rs4804803)	A/A	45 (31.0)	60 (26.0)	1.28 (0.79-2.08)	0.29
	A/G	85 (58.6)	117 (50.6)	1.38 (0.89-2.15)	0.14
	G/G	15 (10.4)	54 (23.4)	0.38 (0.19-0.72)	0.002
		**African American**			
		**SCD**: ***n*** **=** **331** (%)	**Controls**: ***n*** **=** **379** (%)	**Odds ratio** **(95% CI)**	*P*-**value**
CD209 (rs4804803)	A/A	110 (33.2)	124 (32.7)	1.02 (0.74–1.42)	0.94
	A/G	171 (51.7)	191 (50.4)	1.05 (0.77–1.43)	0.76
	G/G	50 (15.1)	64 (16.9)	0.88 (0.57–1.33)	0.54

**Table 3:** Allelic frequency of CD209 polymorphisms between sickle cell and control groups. Allelic frequencies of the CD209 gene promoter (rs4803803) polymorphism, determined among African and American sickle cell disease and control groups. Sickle cell disease (HbSS) populations were recruited from Bamako, Mali (African) and Augusta GA (American). Control populations are individuals without sickle cell disease, and were recruited from Mali (African) and Shreveport LA (American). A/G denotes the alleles at the CD209 locus. Odds ratio was calculated by Fisher’s two-tailed exact test. *P* value <0.05 was considered significant.

		African			
Polymorphism	Allele	SCD: *n* = 290 (%)	Controls: *n* = 462 (%)	Odds ratio (95% CI)	*P*-value
CD209 (rs4804803)	A	175 (60.3)	237 (51.3)	1.44 (1.60–1.97)	0.02
	G	115 (39.7)	225 (48.7)	0.69 (0.51–0.94)	0.02
		**African American**			
		**SCD**: *n* = 662 (%)	**Controls**: *n* = 758 (%)	**Odds ratio** (**95**% **CI**)	*P*-value
CD209 (rs4804803)	A	391 (59.1)	439 (57.9)	1.05 (0.84–1.30)	0.67
	G	271 (40.9)	319 (42.1)	0.95 (0.77–1.19)	0.67

**Notes.**

SCD sickle cell disease CI confidence interval

Since clinical manifestation of sickle cell disease varies greatly within an individual, across individuals of the same population and those of different populations, we evaluated the diversity of CD209 (snp 336A/G) gene promoter polymorphisms between sickle cell groups recruited from Africa and United States. Surprisingly, there was no difference either in genotypic (*P* = 0.19) or allelic frequencies (*P* = 0.72) of mutant variants (snp 336G/G) between sickle cell disease groups (Table 4). The similarities in the genotypic and allelic frequencies (10.4% versus 15.1% and 39.7% versus 40.9% for genotypes and alleles respectively) of the homozygote mutant variants were statistically insignificant.

**Table 4:** Genotypic and allelic frequency of CD209 polymorphisms between sickle cell disease groups. Genotypic and allelic frequencies of the CD209 gene promoter (rs4803803) polymorphism between African and American sickle cell disease groups. Sickle cell disease (HbSS) populations were recruited from Bamako, Mali (African) and Augusta GA (American). A/G denotes the alleles at the CD209 locus. Odds ratio was calculated by Fisher’s two-tailed exact test. *P* value <0.05 was considered significant.

Genotypic frequency					
Polymorphism	Genotype	Mali: *n* = 145 (%)	USA: *n* = 331 (%)	Odds ratio (95% CI)	*P*-value
CD209 (rs4804803)	A/A	45 (31.0)	110 (33.2)	0.90 (0.59–1.40)	0.67
	A/G	85 (58.6)	171 (51.7)	1.32 (0.87–2.00)	0.16
	G/G	15 (10.4)	50 (15.1)	0.65 (0.33–1.23)	0.19
**Allelic frequency**					
**Polymorphism**	**Allele**	**Mali**: ***n*** **=** **290** (%)	**USA**: ***n*** **=** **662** (%)	**Odds ratio** (**95**% **CI**)	***P***-**value**
CD209 (rs4804803)	A	175 (60.3)	391 (59.1)	1.05 (0.79–1.41)	0.72
	G	115 (39.7)	271 (40.9)	0.95 (0.71–1.27)	0.72

**Notes.**

SCD sickle cell disease CI confidence interval

## Discussion

Sickle cell disease is the most commonly inherited hemoglobinopathy with a worldwide distribution. It is a major disease represented in populations of sub-Saharan Africa, the Middle East and several parts of India; it remains a significant health burden borne by the African American population in the United States, as well as in several Caribbean island nations, whose populations are dominated by ethnicities of African origin. It has recently been classified as a disease that would create a global challenge to the population of three major countries (Piel et al., 2013), therefore requiring a need to clarify and decipher the various parameters contributing to its severity and diverse clinical pathophysiology among and between individuals from different populations. To our understanding, this is the first report to elucidate the genomic diversity of CD209 gene promoter (snp-336A/G) polymorphisms in sickle cell disease, with the potential to clarify its role or otherwise in susceptibility to infectious pathogens between sickle cell disease and control groups. We chose three ethnically distinct populations (Bambarans from Mali, African Americans from Shreveport, Louisiana and Augusta, Georgia, and Caucasians from Shreveport Louisiana), and as such permits conclusive inferences based on our finding. African case and controls were all from the Bambaran tribe in Mali, thereby facilitating analysis from an ethnically homogeneous population in comparison to the genetic heterogeneity of the African American group.

Our observation that the CD209 gene promoter homozygous wild-type variant (snp-336A/A) occurred at a lower frequency among Africans compared to African Americans and Caucasians is significant, though not unexpected considering the degree of genetic admixture in the African American population. This is similar to our previous finding while examining the genomic diversity of endothelial nitric oxide synthase gene polymorphisms in differing populations (Thomas et al., 2013). Though both populations share a common ancestry, the many years of genetic admixture and the legacies of slavery would affect the genetic contribution of African genes into the African American genome. The homozygote wild type variant is necessary for dendritic cell activation and initiation of adaptive immune response. Therefore, the reduced frequency of this allele among Africans might be a probable contributory factor to the susceptibility to infectious pathogens. On the other hand, selective pressure would favor those with adapting mechanisms, leading to them becoming adapted to other types of infections, diseases or conditions. A very good example is the hypothesis that sickle cell disease is highly prevalent in malaria endemic areas because of selection pressure that favors individuals with hemoglobin S, believed to be a contributor to malaria resistance in this group (Gong et al., 2012; Gong et al., 2013). Unfortunately, sub-Saharan Africa is blessed with a geographic and weather pattern that sustains the endemicity of many neglected diseases, and could potentially explain the often-encountered cases of multiple co-morbidities in a single host. This could be a disadvantage in the African continent, whereby immune response is limited, contributing to preponderance of infections. The possibility that these infectious agents might have contributed to the imposition of selection pressure (presenting as an advantage among sickle cell disease cases in Mali) is of potential significance and deserves further analysis. One approach to clarify this would be to generate monocyte-derived dendritic cells from peripheral blood mononuclear cells, collected from healthy controls that are CD209 homozygote wild type and homozygote mutant, and between sickle cell disease groups, and evaluate the differential expression of CD14 (monocyte/macrophage marker) or DC-SIGN (dendritic cell marker). A decrease in CD209 expression in the homozygote mutant variant of DC-SIGN in sickle cell disease patients resulting in lower susceptibility to infectious stimulus, with the reverse the case among control groups with the homozygote wild type variant would be a confirmatory outcome. Similar observation has been reported in dengue virus infection (Sakuntabhai et al., 2005; Wang et al., 2011).

An additional mechanism for our observation could be that there is an increased mortality of sickle cell disease patients that are also carriers of the CD209 ‘G’ allele, especially homozygotes, and are therefore missing from the Malian SCD cohort. Such phenomenon might not be the case in Western industrialized countries, where early sickle cell disease mortality is prevented by antibiotics and other prophylactic measures. An alternative approach to decipher the present observation would be to replicate this study in newborns or children with sickle cell disease recruited during the first year of life, before the expected mortality.

This observation in Africans is enhanced by the reverse observation in the Caucasian population of the United States. The wild type variants (AA, AG) allele is 97% among Caucasians and 83% among African Americans, with the mutant variant almost absent in both groups (3.2% among Caucasians and 16% among African Americans). This low genotypic frequency of the homozygote mutant variant is similar to results from previous reports, which showed 0%, 3% and 5% in a Taiwanese, general Brazilian and Sao Paulo populations respectively (Kashima et al., 2009; Wang et al., 2011; Dettogni et al., 2013). In a study conducted among three groups of healthy control populations in Thailand, a similar scenario was observed, with a genotypic frequency of 5%, 1% and 3% (Sakuntabhai et al., 2005). This observation confirms our hypothesis that this marker may have undergone evolutionary change in extant populations outside of Africa Miller, 1994; Gibbons, 2001; Zimmer, 2001; Thomas et al., 2005. Populations with the homozygote wild type variant are able to fight infections, hence the reduced prevalence of infectious agents, while the reverse may be the case in Africa. Further studies are imperative, before a definitive argument can be made, whereby other infectious diseases are examined viz-a-viz genotypic and allelic diversities of CD209 gene promoter polymorphism in the African population. The ancestral-susceptibility model, which states that disease susceptibility alleles are ancestral while derived variants are protective, has been proposed and validated (Di Rienzo & Hudson, 2005; Biswas & Akey, 2006), further emphasizing that ancestral alleles previously adapted might become maladaptive due to dispersal into new environmental niches (Biswas & Akey, 2006). Extensive reports of geographically restricted selection have been found in genome-wide studies of humans and human diseases (Carlson et al., 2005; Weir et al., 2005; Voight, Wen & Pritchard, 2006; Nakajima et al., 2004; Sakagami et al., 2004; Di Rienzo & Hudson, 2005; Young et al., 2005), and seems clear therefore, that local adaptation in extant populations is a major contributor (Fullerton et al., 2002; Rockman et al., 2004; Thompson et al., 2004).

The lack of differences in genotypic and allelic frequencies of homozygote mutant variants between sickle cell disease and control groups in the United States could be due to the low frequency of the mutant allele (small proportion of individuals with the mutant allele) in the US population. A proposed method to clarify this further would be to study sickle cell disease patients from other regions of the United States, considering the known reports of sub-continental regional population substructure in African American genetic makeup (Kayser et al., 2003; Lao et al., 2010) and different rates of Caucasian gene contribution to the genomic ancestry of African Americans.

Based on our present observation, we conclude that the sickle cell gene (as confirmed for malaria infection) probably confers protection against common infectious co-morbidities in Africa. The higher frequency of CD209 gene promoter homozygote mutants in the non-SCD group reveals an impaired capacity to mount an immune response to infectious diseases, potentially a contributor to the dominance of infectious co-morbidities in this population. The CD209 gene promoter polymorphism might be a major player in susceptibility to common infectious pathogens among Africans, and a contributor to diversity and severity of SCD that requires elucidation, while characterizing genetic risks imposed by locale-specific allele frequencies (Mtatiro et al., 2014). The implication of this finding for infectious co-morbidities or as modifiers of SCD pathophysiology, and its significance in African Americans with SCD deserves further deconvolution. Determining if this protection is regulated in any fashion by sickle cell disease haplotypes in Africa (Benin, Bantu, Cameroon, Senegal) and evaluating plasma levels of immunoglobulin E and eosinophilia, as markers of common helminthic infections, between disease and control groups, is needed.

Finally, it is important to clarify the synergistic or pathogenic role of the sickle cell gene in disparate disease and population groups. This report should be considered preliminary because of sample size limitations, thereby advocating for expansive studies in other population groups, as well as examination of other immunogenetic markers, especially as it relates to clinical endpoints in sickle cell disease. Genetic ancestry studies that might clarify the extent of admixture in the American sickle cell disease group and how this impact our current finding would be imperative. Analyzing American sickle cell disease groups, recruited from different regions (Northeast, Mid-Atlantic, Midwest etc.) under same conditions as this report, would be very important, considering the richness and diversity of the African American gene pool (Collins-Schramm et al., 2002; Kittles et al., 2002; Kayser et al., 2003; Lind et al., 2007).

## Supplemental Information

10.7717/peerj.799/supp-1 Supplemental Information 1 Gel electrophoresis of CD209 gene promoter (SNP −336 A/G; rs4804803) amplified PCR products Genomic DNA samples were amplified with standard primers (Dettogni et al., 2013, modified from Sakuntabhai et al.2008); amplified products were digested with *MscI* restriction endonuclease (New England Biolabs), and expressed on a 2% (w/v) ethidium bromide-stained agarose gel. Homozygous wild type variant showed no digestion (150 bp), while mutant variant produced two bands (131 and 19 bp) on digestion; lower band size not seen. Lane 1: 100 bp TriDye DNA ladder (New England Biolabs); lane 2–5: representative homozygous wild type (snp-336 A/A); lane 6–8: representative heterozygote (snp-336 A/G); lane 9–10: representative homozygous mutant (snp-336 G/G)

## Abbreviations

SCD: sickle cell disease
HbAA: hemoglobin AA
HbSS: hemoglobin SS;
PCR-RFLP: polymerase chain reaction-restriction fragment length polymorphism
OR: odds ratio
