# Supplementary figures and images for: Interethnic diversity of the CD209 (rs4804803) gene promoter polymorphism in African but not American sickle cell disease
Source: PeerJ. 2015 Feb 24;3:e799. doi: 10.7717/peerj.799 (PMC4349147; doi:10.7717/peerj.799)

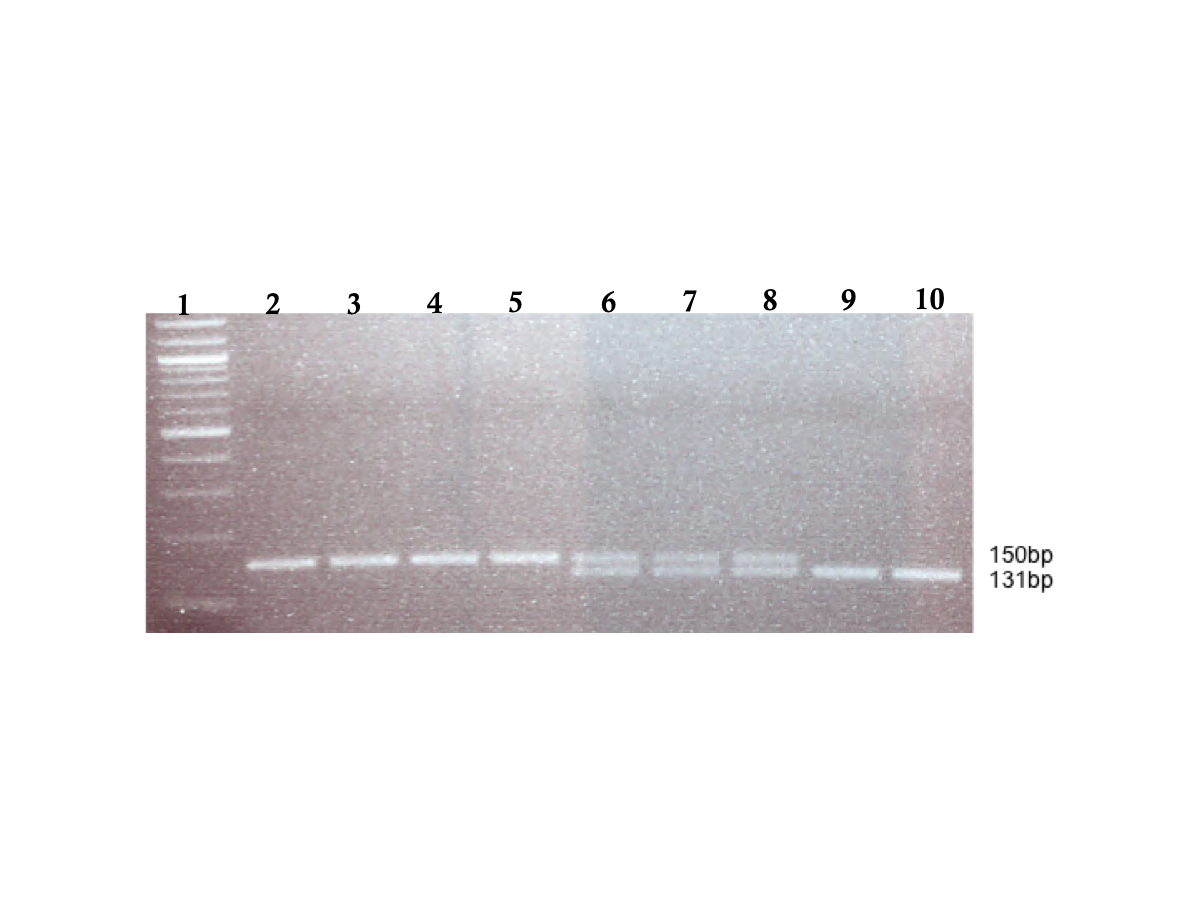

Supplement: Supplemental Information 1 — Genomic DNA samples were amplified with standard primers (Dettogni et al., 2013, modified from Sakuntabhai et al.2008); amplified products were digested with MscI restriction endonuclease (New England Biolabs), and expressed on a 2% (w/v) ethidium bromide-stained agarose gel. Homozygous wild type variant showed no digestion (150 bp), while mutant variant produced two bands (131 and 19 bp) on digestion; lower band size not seen. Lane 1: 100 bp TriDye DNA ladder (New England Biolabs); lane 2–5: representative homozygous wild type (snp-336 A/A); lane 6–8: representative heterozygote (snp-336 A/G); lane 9–10: representative homozygous mutant (snp-336 G/G) [file peerj-03-799-s001.png]
